# Monomelic amyotrophy: a rare disease with unusual features (Hirayama disease)

**DOI:** 10.11604/pamj.2022.42.48.29515

**Published:** 2022-05-18

**Authors:** Chaitanya Kulkarni, Waqar Mohsin Naqvi

**Affiliations:** 1 Community Physiotherapy Department, Ravi Nair Physiotherapy College, Datta Meghe Institute of Medical Sciences, Sawangi (M), Wardha, India,; 2 NKP Salve Institute of Medical Sciences and Research Center, Nagpur, India

**Keywords:** Monomelic amyotrophy, Hirayama disease, UMN and LMN features

## Image in medicine

A 36 years old female presented with chief complains of right sided upper limb weakness which was gradual in onset and progressive in nature. When detailed history was taken, it was observed that she was also having complains of altered sensations and hyperhidrosis in both the palms. On further assessment we found that there was no history of trauma to cervical region or any cervical pain. She has no other past history such as allergies or any exposure to toxins. On neurological assessment we found that patient has muscle atrophy with increased tone in upper limb along with tremors (A) and uncertain atrophy like changes in cervical spine on MRI (Magnetic Resonance Imaging) (B). On further examination clonus was present over left calf with Babinski sign positive. When further investigations were done it was found out that patient has lower and upper motor neuron lesions.

**Figure 1:**
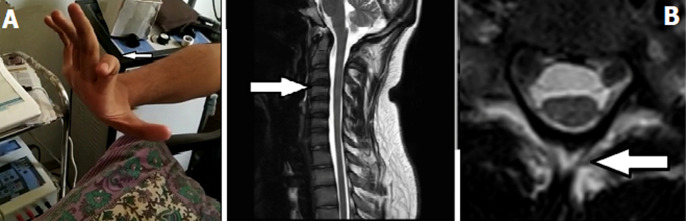
clinical as well as radiological features A) atrophy of the forearm leads to the difficulty of the movements of the hands; B) MRI findings shows unusual atrophy and changes in the cervical spine

